# Potentially Toxic Metals in the High-Biomass Non-Hyperaccumulating Plant *Amaranthus viridis*: Human Health Risks and Phytoremediation Potentials

**DOI:** 10.3390/biology11030389

**Published:** 2022-03-01

**Authors:** Chee Kong Yap, Aziran Yaacob, Wen Siang Tan, Khalid Awadh Al-Mutairi, Wan Hee Cheng, Koe Wei Wong, Franklin Berandah Edward, Mohamad Saupi Ismail, Chen-Feng You, Weiyun Chew, Rosimah Nulit, Mohd Hafiz Ibrahim, Bintal Amin, Moslem Sharifinia

**Affiliations:** 1 Department of Biology, Faculty of Science, Universiti Putra Malaysia, Serdang 43400 UPM, Selangor, Malaysia; g-37177639@moe-dl.edu.my (A.Y.); wongkoewei@gmail.com (K.W.W.); rosimahn@upm.edu.my (R.N.); mhafiz_ibrahim@upm.edu.my (M.H.I.); 2 Department of Microbiology, Faculty of Biotechnology and Biomolecular Sciences, Universiti Putra Malaysia, Serdang 43400 UPM, Selangor, Malaysia; wstan@upm.edu.my; 3 Laboratory of Vaccines and Biomolecules, Institute of Bioscience, Universiti Putra Malaysia, Serdang 43400 UPM, Selangor, Malaysia; 4 Department of Biology, Faculty of Science, University of Tabuk, Tabuk 741, Saudi Arabia; kmutairi@ut.edu.sa; 5 Faculty of Health and Life Sciences, INTI International University, Persiaran Perdana BBN, Nilai 71800, Negeri Sembilan, Malaysia; wanhee.cheng@newinti.edu.my; 6 Natural Resources and Environment Board, Petra Jaya, Kuching 93050, Sarawak, Malaysia; franklin_dwrd@yahoo.com; 7 Fisheries Research Institute, Batu Maung 11960, Pulau Pinang, Malaysia; saupi@rocketmail.com; 8 Department of Earth Sciences, National Cheng-Kung University, No 1, University Road, Tainan City 701, Taiwan; cfy20@mail.ncku.edu.tw; 9 Centre for Pre-University Study, Level, 6, Unity Building, MAHSA University, Bandar Saujana Putra, Jenjarom 42610, Selangor, Malaysia; chewweiyun@gmail.com; 10 Fisheries and Marine Science Faculty, Universitas Riau, Pekanbaru, Riau 28292, Indonesia; bintalamin@gmail.com; 11 Shrimp Research Center, Iranian Fisheries Science Research Institute, Agricultural Research, Education and Extension Organization (AREEO), Bushehr 7516989177, Iran; moslem.sharifinia@yahoo.com

**Keywords:** potentially toxic metals, health risks, phytoremediation, biomonitor

## Abstract

**Simple Summary:**

The goal of this study was to measure the concentrations of four potentially toxic metals in green amaranth (leaves, stems, and roots) collected from 11 sampling sites in Peninsular Malaysia. The danger of the metal concentrations to human health was assessed. The metal levels were highest in the root parts, followed by stems and leafy parts. The positive relationships of metals between plant parts and the habitat topsoils suggested that the green amaranth could be used as a useful biomonitoring agent of Cd, Fe, and Ni pollution. In addition, the green amaranth was also a very promising phytoextraction agent of Ni and Zn and a very promising phytostabiliser of Cd and Fe. This indicates that green amaranth can be used in the phytoremediation of the polluted soils by Cd, Fe, Ni, and Zn. The human health risk assessment for the potentially toxic metals (PTMs) in the leaves of green amaranth indicated that the four metals posed no non-carcinogenic dangers to consumers. However, it is of utmost importance to monitor PTMs in *Amaranthus* fields periodically.

**Abstract:**

Human health risk and phytoremediation of potentially toxic metals (PTMs) in the edible vegetables have been widely discussed recently. This study aimed to determine the concentrations of four PTMs, namely Cd, Fe, Ni, and Zn) in *Amaranthus viridis* (leaves, stems, and roots) collected from 11 sampling sites in Peninsular Malaysia and to assess their human health risk (HHR). In general, the metal levels followed the order: roots > stems > leaves. The metal concentrations (µg/g) in the leaves of *A. viridis* ranged from 0.45 to 2.18 dry weight (dw) (0.05–0.26 wet weight (ww)), 74.8 to 535 dw (8.97–64.2 ww), 2.02 to 7.45 dw (0.24–0.89 ww), and 65.2 to 521 dw (7.83–62.6 ww), for Cd, Fe, Ni, and Zn, respectively. The positive relationships between the metals, the plant parts, and the geochemical factions of their habitat topsoils indicated the potential of *A. viridis* as a good biomonitor of Cd, Fe, and Ni pollution. With most of the values of the bioconcentration factor (BCF) > 1.0 and the transfer factor (TF) > 1.0, *A. viridis* was a very promising phytoextraction agent of Ni and Zn. Additionally, with most of the values of BCF > 1.0 and TF < 1.0, *A. viridis* was a very promising phytostabiliser of Cd and Fe. With respect to HHR, the target hazard quotients (THQ) for Cd, Fe, Pb, and Zn in the leaves of *A. viridis* were all below 1.00, indicating there were no non-carcinogenic risks of the four metals to consumers, including children and adults. Nevertheless, routine monitoring of PTMs in *Amaranthus* farms is much needed.

## 1. Introduction

It is a public health concern that contaminated wastewater was used to irrigate *Amaranthus*, which is a food source for humans in Nigeria [1,2]. The major source of contamination by potentially toxic metals (PTMs) in agricultural lands is usually anthropogenic inputs such as sewage sludge [3] and residues from widespread mining and various industries [4,5]. Improper applications of fertilisers or pesticides from atmospheric sources elevated the concentration of PTMs in soil [6]. In general, the main sources of PTMs are from the manufacturing industries, urbanisation practices, and agro-based industries [7] due to the effects of wastewater used for the irrigation of *Amaranthus* have been reported in the literature [1,2,8].

The present study investigated the presence of PTMs in *A. viridis* in Malaysia, which is considered a high-biomass non-hyperaccumulating plant (HBNP). Suman et al. [9] reported that the metal limits in the dry biomass of plants to be considered as hyperaccumulators are 1.00 × 10^2^ mg/kg for Cd, 1.00 × 10^3^ mg/kg for Ni, and 1.00 × 10^4^ mg/kg for Zn [10]. These values are up to 100–1000-fold more than non-hyperaccumulating species under comparable conditions [11,12].

The use of fast-growing weeds and non-woody species for phytoextraction is a sensible decision, owing to the fact that these taxa (i) can provide high biomass in a moderately short time frame, (ii) have a high rate of water uptake and a deep root framework, and (iii) can be adequately and quickly reused through re-growing, and (iv) their aboveground parts are not difficult to sample. Restorative plants such as peppermint (*Mentha piperita*) and lavender (*Lavandula angustifolia*) have been demonstrated to accumulate elevated levels of PTMs in their biomass [13,14]. For example, maize (*Zea mays*) is generally known as HBNP for its quick growth rate, high biomass production, and general Cd-resistance. In a field-scale preliminary study on Cd-polluted farmland in China, *Z. mays* was found to accumulate up to 3 mg/kg of dry biomass while keeping grain Cd levels under the Chinese government’s breaking point for coarse oats [15].

Kanakaraju et al. [16] studied the accumulations of Zn, Cu, Mn, Co, Pb, and Fe in leafy vegetables (kala, green mustard, and white mustard), as well as the long bean gathered from Siburan and Beratok at Kuching, Sarawak, Malaysia. They found marginally higher Pb ranges in the vegetables. Molina et al. [17] reported the presence of nine PTMs (including Cd, Fe, and Zn) in *A. dubius* used as forage in the diet of sheep, goats, pigs, and cattle. Studies on PTMs health risk assessments and phytoremediation capacity of edible *Amaranthus* have been reported in literature [18,19,20,21] but, to our knowledge, any specific trial on *A. viridis* has not been done.

A plant potential as a phytoremediator can be determined by calculating the plant bioconcentration factor (BCF; metal concentration ratio of plant roots to the soil) and translocation factor (TF; metal concentration ratio of plant shoots to roots) values [22]. The BCF (remainder of the substance of a given metal of the plant to its substance of soil) characterises the capacity of the plant to accumulate PTMs. The TF is a proportion of the phytoextraction limit of plants [23]. If both the BCF and TF values are above 1, the plant species has the potential to be a phytoextractor of metals, while a BCF value above 1 but a TF value below 1, indicating that the plant has potential as a phytostabiliser of metals [24].

In this study, *A. viridis* was studied because (i) it is among the top ten most popular vegetables, which is easy to grow and can quickly be harvested just after 28 days; (ii) it has been used in previous research studies and reported in literature [3]; and (iii) it is highly recommended by the District Agriculture Department of Hulu Perak (Peninsular Malaysia) as a research vegetable. Therefore, the present study aimed (i) to determine the PTMs levels (Cd, Fe, Ni, and Zn) in *A. viridis* (leaves, stems, and roots) collected from Peninsular Malaysia, (ii) to assess the human health risks of PTMs in the edible leafy parts of *A. viridis* from Peninsular Malaysia, and (iii) to assess the potentials of *A. viridis* as a phytoremediator of PTMs.

## 2. Materials and Methods

### 2.1. Study Area

**Figure 1:**
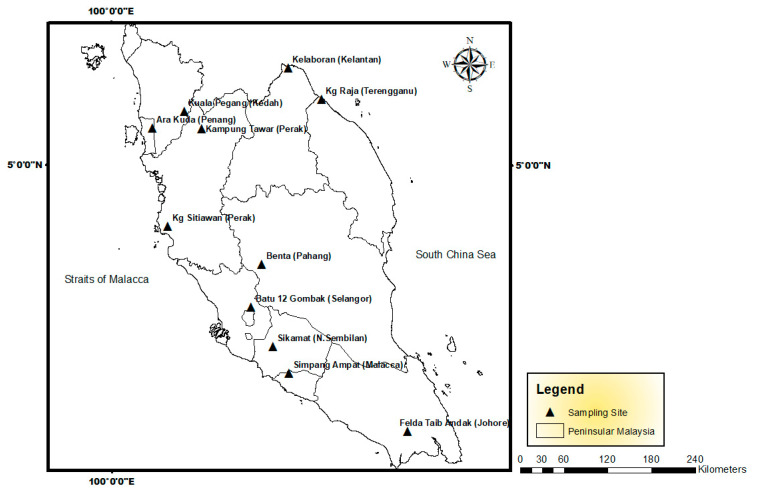
Sampling map for the *Amaranthus viridis* and their habitat topsoils in Peninsular Malaysia.

Samples were collected within 6 months on 11 sites located in Peninsular Malaysia from July 2017 to November 2018 (Figure 1; Table 1).

### 2.2. Sampling and Sample Preparation

At each sampling sites, five plants of *A. viridis* and three replicates of their habitat topsoils were collected at the same time. The 11 different sampling sites included the farm areas that potentially received wastewater for irrigation in Perak and Penang (Table 1). Additionally, these sites also included those in the vicinity of mining, industrial, landfill, agricultural, and residential areas. Five subsamples were collected from each sampling site. At the same time, three replicates of habitat topsoils (0–10 cm) were sampled. All collected samples of *A. viridis* and topsoils were stored in clean polythene bags. Later, they were transported to the laboratory at Universiti Putra Malaysia, Serdang, Malaysia for further analyses.

The collected samples were washed with distilled water to remove dust particles. The *A. viridis* samples were separated into stems, roots, and leaves by cutting them into small pieces using a clean knife. Different parts (roots, stems, and leaves) of *A. viridis* were dried in an oven at 60 °C, for at least three days. After drying, the separated parts of vegetable samples were ground into a fine powder using a commercial blender and stored in polyethylene bags until they were used for acid digestion.

The gathered topsoil samples were dried in a stove at 100 °C. After drying, the dried soils were crushed into fine powder utilising a mortar and pestle, and later they were sieved through a 63 µm mesh strainer.

### 2.3. Digestion of Plant Samples

The aqua-regia technique was applied for the extraction of metals in the plant samples. A total of 5 ml of concentrated nitric acid (HNO_3_, AnalaR grade, BDH 69%) were added to the dried tissues. Each dried soil sample (1.0 g) was digested using a combination of concentrated nitric acid (HNO_3_, AnalaR grade, BDH 69%) and perchloric acid (HClO_4_, AnalaR grade, BDH 60%) in the ratio 4:1 (10 mL) [25].

Samples were then placed in a digestion block at 40 °C for 1 h, and the samples were then fully digested at 140 °C for 3 h [25]. They were then diluted to 40 mL with double de-ionised water. Later, the diluted samples were filtered through Whatman No. 1 (filter speed: medium) filter paper into acid-washed pill boxes and stored at 4 °C until metal determination.

The topsoils were fractionated into four fractions based on Badri and Aston [26,27] and Wong et al. [28]. These four fractions employed in this study were (i) ‘Easily, freely, leacheable, or exchangeable’ (EFLE); (ii) ‘Acid-reducible’ (AR); (iii) ‘Oxidisable–organic’ (OO); and (iv) ‘Resistant’ (RES).

### 2.4. Metal Analysis

All the samples were analysed for Cd, Fe, Ni, and Zn by using an air-acetylene flame atomic absorption spectrophotometer (FAAS, Perkin Elmer Model AAnalyst 800; Perkin Elmer LLC, CT, USA). Standard solutions were prepared from 10,000 ppm stock solution provided by MERCK Titrisol for the six metals [25], and the data were presented in μg/g dry weight basis.

### 2.5. Quality Control and Quality Assurance

All glassware and non-metal apparatuses used in this study were soaked in an acid bath (5% HNO_3_) for 72 h after being washed with laboratory grade detergent (Decon 90), to avoid possible contamination. The metal-made apparatuses were washed and soaked in laboratory-grade detergent (Decon 90) for at least 3 h before the analysis. Procedural blanks were employed, and quality control samples were made by diluting the standard solutions of the metals to be tested. These standard solutions were analysed after every 5–10 samples in order to check for the accuracy of the analysed samples.

Four types of Certified Reference Materials (CRMs) were checked with the samples to ensure the accuracy of the FAAS measurements. These CRMs included *Lagarosiphon major* (NR.60), Dogfish Liver-DOLT-3 (National Research Council Canada), marine sediments-(MESS-3, National Research Council Canada, Beaufort Sea), and NSC DC 73,319 (soil). Their recoveries were mostly acceptable (between 70% and 120%).

### 2.6. Human Health Risk Assessments

For the assessments of health risks, all the metal concentrations formerly presented in dry weight (dw) basis were converted into wet weight (ww) basis by using a conversion factor of 0.12 [29,30,31]. In order to evaluate a once or long-term potential hazardous exposure to metals through consumption of edible vegetables (USEPA, 1989) by the population of Peninsular Malaysia, the estimated daily intake (EDI), and the target hazard quotient (THQ) of PTMs were calculated using the following formulas:

Human health risk (HHR) arose due to consumption of vegetables exposed to toxic metals was evaluated by the mean of the calculation of the EDI and the THQ [32]. The calculation formula for EDI is as follows:

(1) $$\mathrm{EDI}=\frac{\mathrm{Mc}\;\times \mathrm{consumption}\;\mathrm{rate}}{\mathrm{body}\;\mathrm{weight}}$$

where Mc is the metal concentration (µg/g wet weight) in the vegetables. The body weights for children and adults were 17 and 69.2 kg, respectively [33,34], and the consumption rates were 17.0 and 34.0 g/day, for children and adults, respectively [35].

The THQ was calculated based on Equation (2):

THQ= EDI/RfD(2)

The oral reference portion (RfD) was compared with the EDIs (µg/kg wet weight/day) of metals in vegetables. The oral reference portion (RfD) (µg/kg wet weight/day) utilised in this investigation were Cd: 1.00, Fe: 700, Ni, 20.00, and Zn: 300, as given by the EPA’s Integrated Risk Information System online data set (IRIS) [36]. The oral reference portion is the basic grouping of take-up of a metal, under which there would not be any apparent danger [37].

### 2.7. Calculation of Translocation Factor and Bioconcentration Factor

Both the translocation factor (TF) and the bioconcentration factor (BCF) were utilised to calculate the plant’s ability to uptake and withstand metals.

These two indices are commonly used to determine the suitability of plants as good phytoremediators. BCF is defined as in Equation (3):

(3) $${\mathrm{BCF}}_{\mathrm{plant}}=\frac{{\mathrm{Plant}}_{\mathrm{metal}}}{{\mathrm{Soil}}_{\mathrm{metal}}}$$

where the plant parts were leaves, stems, or roots.

TF is defined as in Equations (4) and (5):

(4) $${\mathrm{TF}}_{\mathrm{stem}}=\frac{{\mathrm{Stem}}_{\mathrm{metal}}}{{\mathrm{Root}}_{\mathrm{metal}}}$$

(5) $${\mathrm{TF}}_{\mathrm{leaf}}=\frac{{\mathrm{Leaf}}_{\mathrm{metal}}}{{\mathrm{Root}}_{\mathrm{metal}}}$$

### 2.8. Statistical Analysis

In order to reduce the variance [38], the Pearson’s correlation analysis (CA) and stepwise multiple linear regression analysis (SMLRA) were based on log_10_ transformed data of the metals using the STATISTICA (Version 10; StatSoft. Inc., Tulsa, OK, USA, 1984–2011). After the log_10_ transformation on the data of Cd, Fe, Ni, and Zn, the plants and the topsoils showed that all the data were within the normality ranges for skewness (−2 to +2) [39,40]. Differences of metal concentrations in the different parts of the plants were analysed by using the post-hoc test (Student–Newman–Keuls) in the one-way ANOVA analysis to see if there was any significant difference at *p* < 0.05. This post-hoc test was performed by using SPSS Statistics for Windows, version 18.0 (SPSS Inc., Chicago, IL, USA).

Both CA and SMLRA were used to see the relationships of metal concentrations between the different parts in the plants and geochemical factions in the habitat topsoils. The general purpose of the SMLRA is to find the most influential independent variables (represented by the metal concentrations in the geochemical fractions in the habitat topsoils) that could influence the dependent variables (represented by metal concentrations in the different parts of plants).

## 3. Results

### 3.1. Potentially Toxic Metals Concentrations in Amaranthus

**Figure 2:**
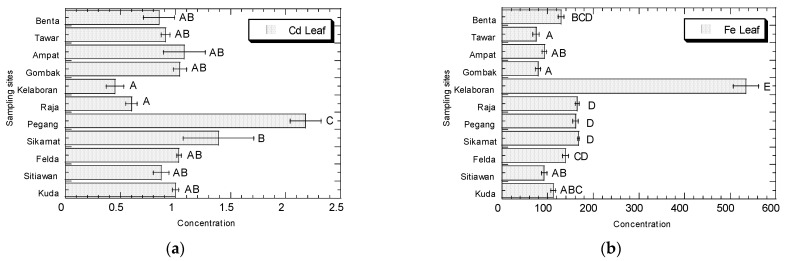

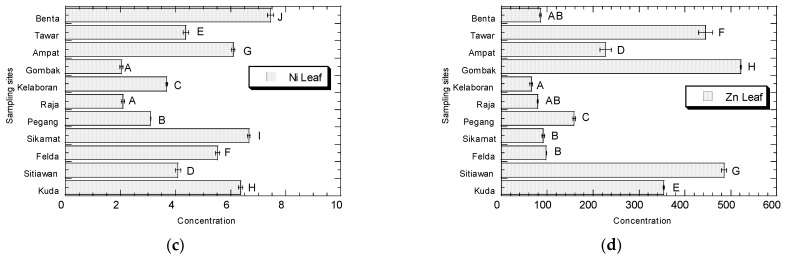
Concentrations (mean ± SE, µg/g dry weight) of Cd (**a**), Fe (**b**), Ni (**c**), and Zn (**d**) in the leaves of *Amaranthus viridis* collected from all the sampling sites in Peninsular Malaysia. Metal concentrations sharing a common letter are not significantly different (*p* > 0.05) based on the post-hoc test (Student–Newman–Keuls) in the one-way ANOVA analysis.

**Figure 3:**
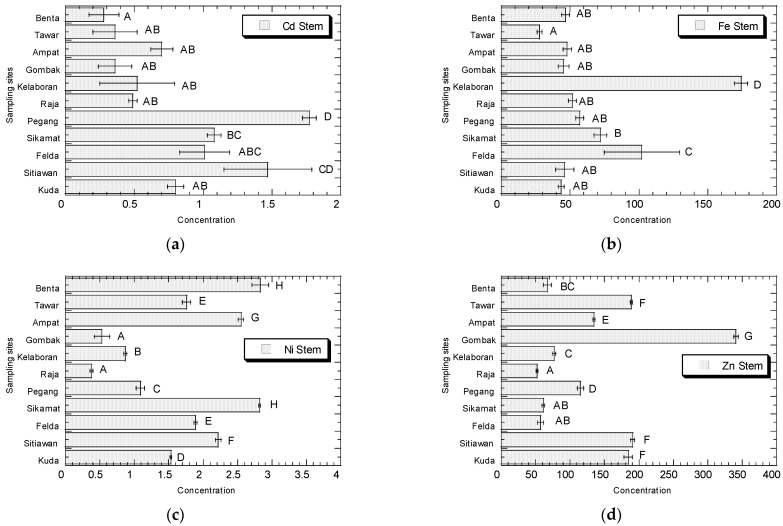
Concentrations (mean ± SE, µg/g dry weight) of Cd (**a**), Fe (**b**), Ni (**c**), and Zn (**d**) in the stems of *Amaranthus viridis* collected from all the sampling sites in Peninsular Malaysia. Metal concentrations sharing a common letter are not significantly different (*p* > 0.05) based on the post-hoc test (Student–Newman–Keuls) in the one-way ANOVA analysis.

**Figure 4:**
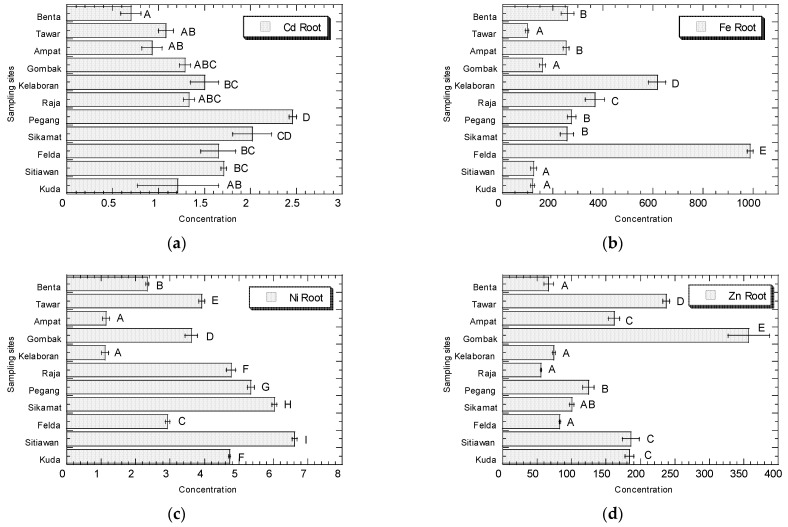
Concentrations (mean ± SE, µg/g dry weight) of Cd (**a**), Fe (**b**), Ni (**c**), and Zn (**d**) in the roots of *Amaranthus viridis* collected from all the sampling sites in Peninsular Malaysia. Metal concentrations sharing a common letter are not significantly different (*p* > 0.05) based on the post-hoc test (Student–Newman–Keuls) in the one-way ANOVA analysis.

The concentrations (µg/g dry weight) of Cd, Fe, Ni, and Zn in the leaves, stems, and roots of *A. viridis* collected from Peninsular Malaysia are presented in Figure 2, Figure 3 and Figure 4, respectively.

For the leaves, Kuala Pegang had the highest levels of Cd (2.18), Kelaboran showed the highest Fe concentration (535), while Benta and Sikamat had the highest concentrations of Ni (7.45 and 6.67, respectively). Kg Sitiawan and Bt 12 Gombak showed the higher concentrations of Zn (521 and 485, respectively). In general, Kuala Pegang showed the highest concentrations of Cd.

For the stems, the samples from Kuala Pegang and Kg Sitiawan showed the highest concentrations of Cd (1.77 and 1.47, respectively). Higher levels of Fe were found in the stems at Kelaboran (174) and Felda Taib Andak (102). Higher levels of Ni were found at Sikamat, Benta, Simpang Ampat, and Kg Sitiawan (2.81, 2.80, 2.54, and 2.21, respectively). Higher levels of Zn were found at Bt 12 Gombak, Kg Sitiawan, and Ara Kuda (341, 191, and 184, respectively).

For the roots, Kuala Pegang and Sikamat had the highest concentrations of Cd (2.46 and 2.02, respectively). Felda Taib Andak and Kelaboran showed the highest Fe concentrations (981 and 617, respectively) when compared to the other sites. Kg Sitiawan, Sikamat, and Kuala Pegang showed higher levels of Ni (6.61, 6.02, and 5.34, respectively). Bt 12 Gombak and Kg Tawar had the higher levels of Zn (357 and 238, respectively). In general, Sikamat, Felda Taib Andak, Kelaboran, and Kg Tawar had higher concentrations of PTMs in roots when compared with the other sites.

In leaves, the Cd concentrations ranged from 0.45 to 2.18 µg/g dw (0.05–0.26 µg/g ww). The Fe concentrations ranged from 74.8 to 535 µg/g dw (8.97–64.19 µg/g ww). The Ni concentrations ranged from 2.02 to 7.45 µg/g dw (0.24–0.89 µg/g ww). The Zn concentrations ranged from 65.2 to 521 µg/g dw (7.83–62.57 µg/g ww) (Table S1).

In stems, the Cd concentrations ranged from 0.28 to 1.77 µg/g dw (0.02–0.11 µg/g ww). The Fe concentrations ranged from 27.72 to 174.17 µg/g dw (1.66–10.45 µg/g ww). The Ni concentrations ranged from 0.37 to 2.81 µg/g dw (0.02–0.17 µg/g ww). The Zn concentrations ranged from 52.15 to 341.1 µg/g dw (3.13–20.47 µg/g ww) (Table S1).

In roots, the Cd concentrations ranged from 0.70 to 2.46 µg/g dw (0.08–0.27 µg/g ww). The Fe concentrations ranged from 101.64 to 987.59 µg/g dw (11.18–108.63 µg/g ww). The Ni concentrations ranged from 1.12 to 6.61 µg/g dw (0.12–0.73 µg/g ww). The Zn concentrations ranged from 55.71 to 357.20 µg/g dw (6.13–39.29 µg/g ww) (Table S1).

### 3.2. Metal Levels in the Habitat Topsoils of Amaranthus

The concentrations of Cd, Fe, Ni, and Zn in the geochemical fractions of the habitat topsoils of *A. viridis* collected from all sampling sites in Peninsular Malaysia are presented in Tables S2, S3, S4, and S5, respectively. The overall concentrations of Cd, Fe, Ni, and Zn in the geochemical fractions of the habitat topsoils of *A. viridis* collected from Peninsular Malaysia are presented in Table 2.

The Cd concentrations (µg/g dw) in the EFLE, AR, OO, RES, and SUM in the habitat topsoils of *A. viridis* ranged from 0.18 to 0.45, 0.24 to 0.71, 0.26 to 1.33, 1.12 to 4.00, and 2.11 to 5.17, respectively (Table S2). The Fe concentrations (µg/g dw) in the EFLE, AR, OO, RES, and SUM in the habitat topsoils of *A. viridis* ranged from 0.40 to 1.39, 26.5 to 216, 105 to 244, 8084 to 58,216, and 8430 to 58,375, respectively (Table S3). The Ni concentrations (µg/g dw) in the EFLE, AR, OO, RES, and SUM in the habitat topsoils of *A. viridis* ranged from 0.25 to 1.70, 0.20 to 4.27, 2.43 to 9.53, 2.94 to 14.4, and 8.07 to 24.8, respectively (Table S4). The Zn concentrations (µg/g dw) in the EFLE, AR, OO, RES, and SUM in the habitat topsoils of *A. viridis* ranged from 1.38 to 5.90, 10.7 to 57.6, 15.8 to 93.5, 14.8 to 117, and 42.7 to 246, respectively (Table S5).

### 3.3. Correlations of Metals between Amaranthus and the Geochemical Fractions of the Habitat Topsoils

The correlation coefficients of metal concentrations between *A. viridis* (leaves, stems, and roots) and their habitat topsoils are presented in Table 3, while SMLRA results are presented in Table 4.

When the correlation results (Table 3) were compared to those of SMLRA (Table 4), it was found that the Cd leaf was significantly (*p* < 0.05) influenced by EFLE and OO based on the SMLRA result. The Cd stem was significantly (*p* < 0.05) influenced by RES and EFLE, while the Cd root was significantly (*p* < 0.05) influenced by SUM. Thus, the Cd SMLRA result complemented the significant correlation results, in which significant (*p* < 0.05) and positive correlations were found in the pairwises for Cd leaf–EFLE, Cd stem–RES, Cd stem–SUM, Cd root–RES, and Cd root–SUM.

For Fe, significant (*p* < 0.05) and positive correlations were found in the pairwises for root–RES and root–SUM, while negative significant (*p* < 0.05) correlations were found for stem–OO and root–AR. Fe–leaf was significantly (*p* < 0.05) influenced by OO, while Fe–stem was significantly (*p* < 0.05) influenced by OO and RES. Lastly, Fe–root was significantly (*p* < 0.05) influenced by RES.

For Ni, significant (*p* < 0.05) and positive correlations were found in the pairwises for leaf–RES and stem–RES. The Ni-leaf was significantly (*p* < 0.05) influenced by RES and OO, while Ni–stem was significantly (*p* < 0.05) influenced by RES. Lastly, the Ni–root was significantly (*p* < 0.05) influenced by EFLE, OO, and AR. Therefore, the accumulations of Cd, Fe, and Ni in the leaves, stems, and roots were influenced by the respective metals in the geochemical fractions of the habitat topsoils. However, there were no significant (*p* > 0.05) correlations for all Zn pairwises. Practically, there were no clear correlations for Zn pairwise. The MLSRA results for Zn, especially in Zn-leaf and Zn-root, did not show significant (*p* > 0.05) selection of Zn geochemical fractions of the habitat topsoils. However, Zn-stem was significantly (*p* < 0.05) influenced by EFLE, OO, and AR.

### 3.4. Human Health Risk of Metals in Amaranthus

The values of EDI and THQ on the edible leaves of *A. viridis* from the present study of the four PTMs for children and adults from all the sampling sites in Peninsular Malaysia are presented in Table S6. Table 5 shows the overall values of EDI and THQ on the edible leaves of *A. viridis*.

The EDI values of Cd for adults and children ranged from 0.06 to 0.29 and 0.03 to 0.13, respectively. The THQ values of Cd for adults and children ranged from 0.059 to 0.285 and 0.027 to 0.129, respectively. The EDI values of Ni for adults and children ranged from 0.26 to 0.97 and 0.12 to 0.44, respectively. The THQ values of Ni for adults and children ranged from 0.013 to 0.049 and 0.006 to 0.022, respectively.

The EDI values of Zn for adults and children ranged from 8.53 to 68.20 and 3.85 to 30.70, respectively. The THQ values of Zn for adults and children ranged from 0.028 to 0.227 and 0.013 to 0.102, respectively. The EDI values of Fe for adults and children ranged from 9.78 to 69.95 and 4.41 to 31.54, respectively. The THQ values of Fe for adults and children ranged from 0.014 to 0.100 and 0.006 to 0.045, respectively. Therefore, the THQ values for Cd, Fe, Ni, and Zn in the *A. viridis* were all below 1.00, indicating that there were no non-carcinogenic risks of the six metals to the consumers, including children and adults.

### 3.5. The Translocation Factor and Bioconcentration Factor of Amaranthus

The values of bioconcentration factors (BCF) of PTMs on the leaves, stems, and roots of *A. viridis* from all the sampling sites in Peninsular Malaysia are presented in Table S7. The overall values of BCF of PTMs on the leaves, stems, and roots of *A. viridis* from Peninsular Malaysia are presented in Table 6. The values of BCF_leaf/EFLE_ for Cd, Fe, Ni, and Zn ranged from 2.06–5.70, 97.09–972.74, 2.13–22.12, and 13.82–205.58, respectively. The values of BCF_leaf/SUM_ for Cd, Fe, Ni, and Zn ranged from 0.10–0.51, 0.00–0.03, 0.14–0.83, and 0.33–5.09, respectively. The values of BCF_stem/EFLE_ for Cd, Fe, Ni, and Zn ranged from 1.02–5.73, 37.20–316.72, 0.51–7.56, and 9.10–80.83, respectively. The values of BCF_stem/SUM_ for Cd, Fe, Ni, and Zn ranged from 0.10–0.37, 0.00–0.01, 0.02–0.35, and 0.22–2.00, respectively. The values of BCF_root/EFLE_ for Cd, Fe, Ni, and Zn ranged from 2.52–7.06, 153.34–1277.18, 0.73–11.72, and 9.72–101.07, respectively. The values of BCF_root/SUM_ for Cd, Fe, Ni, and Zn ranged from 0.24–0.51, 0.00–0.03, 0.05–0.75, and 0.23–2.18, respectively.

The values of TF of PTMs on the leaves, stems, and roots of *A. viridis* from all the sampling sites in Peninsular Malaysia are presented in Table S8. The overall values of TF of PTMs on the leaves, stems, and roots of *A. viridis* in Peninsular Malaysia are presented in Table 7. The values of TF_leaf/root_ for Cd, Fe, Ni, and Zn ranged from 0.30–1.23, 0.14–0.94, 0.44–5.34, and 0.88–2.61, respectively.

The values of TF_stem/root_ for Cd, Fe, Ni, and Zn ranged from 0.28–0.86, 0.10–0.38, 0.08–2.23, and 0.61–1.04, respectively. According to Subha and Srinivas [41], the BCF values were reported as Cd (2.68), Ni (6.80), and Zn (1.27) in the common marsh buckwheat *Polygonum glabrum* collected from The Hussain Sagar Lake, India.

Based on the values of Cd BCF in the leaves (Table S7), all the 11 sampling sites were found with BCF_leaf/EFLE_ > 1.0 but all sites were found with BCF_leaf/SUM_ < 1.0. Based on the values of Cd BCF in the stems (Table S7), all 11 sampling sites were found with BCF_stem/EFLE_ > 1.0, but all sites were found with BCF_stem/SUM_ < 1.0. Based on the values of Cd BCF in the roots (Table S7), all 11 sampling sites were found with BCF_root/EFLE_ > 1.0, but all sites were found with BCF_root/SUM_ < 1.0. This shows that the Cd in the topsoil EFLE fraction could be more easily transferred to the roots, leaves, and stems when compared to those in the topsoil total concentrations of Cd. Based on the values of Cd TF (Table S8), nine sites (82%) were found with TF_leaf/root_ < 1.0, while all sites (100%) with TF_stem/root_ < 1.0 were found. This shows that Cd transfer to the stems from the roots was not efficient in most sampling sites, while Cd transfer to the leaves from the roots was less efficient in all sampling sites. Thus, with most of the values of BCF > 1.0 and TF < 1.0, *A. viridis* has the potential to be used in phytostabilisation of Cd [24].

Based on the values of Fe BCF in the leaves (Table S7), all 11 sampling sites were found with BCF_leaf/EFLE_ > 1.0, but all sites were found with BCF_leaf/SUM_ < 1.0. Based on the values of Fe BCF in the stems (Table S7), all 11 sampling sites were found with BCF_stem/EFLE_ > 1.0, but all sites were found with BCF_stem/SUM_ < 1.0. Based on values of Fe BCF in the roots (Table S7), all 11 sampling sites were found with BCF_root/EFLE_ > 1.0, but all sites were found with BCF_root/SUM_ < 1.0. This shows that the Fe in the topsoil EFLE fraction could be more easily transferred to the roots, leaves, and stems when compared to those in the topsoil total concentrations of Fe. Based on the values of Fe TF (Table S8), all 11 sites (100%) were found with TF_leaf/root_ < 1.0, while all sites (100%) with TF_stem/root_ < 1.0 were also found. This shows that Fe transfer to the stems from the roots was not efficient at all in all sampling sites. Similarly, Fe transfer to the leaves from the roots was also not efficient in all sampling sites. Hence, with all of the values of BCF > 1.0 and TF < 1.0, *A. viridis* has the potential to be used in the phytostabilisation of Fe [24].

Based on the values of Ni BCF in the leaves (Table S7), all 11 sampling sites were found with BCF_leaf/EFLE_ > 1.0, but all sites were found with BCF_leaf/SUM_ < 1.0. Based on the values of Ni BCF in the stems (Table S7), all eight sampling sites (73%) were found with BCF_stem/EFLE_ > 1.0, but all sites were found with BCF_stem/SUM_ < 1.0. Based on values of Ni BCF in the roots (Table S7), all nine sampling sites (82%) were found with BCF_root/EFLE_ > 1.0, but all sites were found with BCF_root/SUM_ < 1.0. This shows that the Ni in the topsoil EFLE fraction could be more easily transferred to the roots, leaves, and stems when compared to those in the topsoil total concentrations of Ni. Based on the values of Ni TF (Table S8), seven sites (64%) were found with TF_leaf/root_ > 1.0, while nine sites (82%) with TF_stem/root_ < 1.0 were found. This shows that Ni transfer to the leaves from the roots was efficient in all sampling sites, while Ni transfer to the stems from the roots was more efficient in most sampling sites. Thus, with most of the values of BCF > 1.0 and TF > 1.0, *A. viridis* is a potential phytoextraction agent of Ni [24].

Based on the values of Zn BCF in the leaves (Table S7), all sampling sites (100%) were found with BCF_leaf/EFLE_ > 1.0, while seven sites (64%) were found with BCF_leaf/SUM_ > 1.0. Based on the values of Zn BCF in the stems (Table S7), all sampling sites (100%) were found with BCF_stem/EFLE_ > 1.0, while six sites (55%) were found with BCF_stem/SUM_ > 1.0. Based on values of Zn BCF in the roots (Table S7), all 11 sampling sites (100%) were found with BCF_root/EFLE_ > 1.0, while seven sites (64%) were found with BCF_root/SUM_ > 1.0. This shows that the Zn in the topsoil EFLE fraction could be more easily transferred to the roots, leaves, and stems when compared to those in the topsoil total concentrations of Zn. Based on the values of Zn TF (Table S8), nine sites (82%) were found with TF_leaf/root_ > 1.0, while four sites (36%) with TF_stem/root_ > 1.0 were found. This shows that Zn transfer to the leaves from the roots was efficient in most sampling sites, while Zn transfer to the stems from the roots was efficient in some sampling sites. Therefore, with most of the values of BCF > 1.0 and TF > 1.0, *A. viridis* is a very promising phytoextraction agent of Zn [24].

## 4. Discussion

### 4.1. Potentially Toxic Metals Concentrations in Amaranthus

The current Cd range (0.05–0.26 µg/g ww) in the edible leaves of *A. viridis* was higher than the maximum permissible limit for Cd (0.05 µg/g ww) for eatable pieces of vegetables set up by China [42]. Yang et al. [43] revealed that the Cd levels (µg/g ww) in eatable vegetables gathered from the six chosen nursery vegetable bases from eastern China were reliably higher in the leafy vegetables (0.009–0.09) than those (0.002–0.017) in the products of the soil from every one of the six bases. The concentrations of Cd in the present study were higher than those reported by Li et al. [44] on the leafy vegetables.

Hu et al. [45] revealed that the mean concentrations (µg/g ww) of Cd in four types of leafy vegetables (Cd: 0.013) were higher than those in the four types of products of the soil: (Cd: 0.005). Fan et al. [46] reported that the concentrations (µg/g ww) of Cd in nursery vegetables were 0.02, while foods grown from the ground were 0.03 for leafy vegetables. As a rule, the pattern of metal exchange in various vegetable types was leaves > soils. The higher groupings of Cd in leaves than those in the soils showed that leafy vegetables had higher capacity for the accumulation of the two metals. The more elevated levels of metals in the leafy vegetables concurred with past discoveries [43]. This demonstrated that leafy vegetables had higher transportation rates than other vegetable types [47]. This could be due to more hindrances, keeping PTM transmission from soil to organic products than those to leaves [48].

According to information from Li et al. [44], the Ni levels (µg/g ww) in the products of the soil were from 0.054 to 0.536 (mean: 0.184). The more significant levels of substantial metals in the leafy vegetables concurred with past discoveries [43,45,46]. This shows that leafy vegetables had higher transportation rates than other vegetable types [47]. This could be due to more obstructions, keeping PTM transmission from soil to natural products than to leaves [48].

The Zn concentrations (7.83–62.57 µg/g ww) in the edible leaves of *A. viridis* were higher than the maximum permissible concentration for Zn (20.0 µg/g ww) for edible parts of vegetables established by China [42]. The mean concentrations of Zn in the leaves of *A. viridis* were below the maximum permissible levels suggested by FAO/WHO [49] (Zn: 60 µg/g ww) for leafy and fruit vegetables. Li et al. [44] reported that the Zn concentrations (µg/g ww) in the leafy vegetables ranged from 1.64 to 5.79.

Hu et al. [45] reported that the mean levels (µg/g ww) of Zn in four types of leafy vegetables (Zn: 3.65) were higher than those in the four types of products of the soil: (0.481; Zn: 1.53). Additionally, Sridhara Chary et al. [50] revealed the higher enhancement factor for PTMs in leafy vegetables. As indicated by Yang et al. [51], movement of PTMs from soil to plants was managed by the root cell divider, the particle transmembrane transport in the endoderm cytoplasm film, and the water transport in the xylem vessel. Contrasted with the stem part, the leaves of *A. viridis* had moderately higher levels of Zn. This shows that the leafy part had a higher danger of PTM accumulation. This outcome concurred with the findings of Hu et al. [52] and Yang et al. [43].

Azi et al. [18] reported concentrated PTM levels in *A. viridis* leaves collected from Abakaliki, Nigeria. They concluded that the *A. viridis* leaves contained unsatisfactory degrees of harmful PTMs and therefore presented a critical wellbeing hazard for the purchasers. Oluwatosin et al. [20] explored the accumulations of Cd and Zn in vegetables (*A. hybridus*) grown in the valley base soils of certain urban communities in southwestern Nigeria. The metal levels in vegetable leaves ranged from 0.38 to 1.20 for Cd and 8.2 to 30.4 for Zn. In the vegetable stems, the PTM levels went from 0.6 to 2.5 for Cd and 11.4 to 18.9 for Zn. Concentrations of metals in vegetable roots went from 1.4 to 4.9 for Cd and 10.2 to 29.0 for Zn. TF values were in the range of 0.22 and 3.00, with Cd having the most elevated TF of 3.00.

Sahu and Kacholi [21] examined the degrees of Zn, Fe, and Co in *Amaranthus* species collected from a neighbourhood garden situated at Chang’ombe-Mchicha region in Temeke District, in the Dar es Salaam locality, Tanzania. The nursery was flooded by wastewater from a stream streaming close by, which flowed through the modern regions prior to arriving at the nursery. The results showed that the leaves had higher metal levels than those in the stems and roots. The normal PTM levels for Zn are 6.87 to 11.59 mg/100 g, and for Fe, 13.40 to 33.65 mg/100 g.

### 4.2. Metal Levels in the Habitat Topsoils of Amaranthus

The present study’s metal levels of the habitat topsoils of *A. viridis* collected from Peninsular Malaysia were comparable to those of most reported studies in the literature. Li et al. [53] investigated the effects of greenhouse cultivation on transport and distribution of Cu, Zn, Fe, Mn, Pb, and Cd in the soil–vegetable system in cucumber (*Cucumis sativus* L.) and tomato (*Lycopersicon esculentum*), between greenhouse and open field cultivations. They reported that the Zn concentrations in the cucumber habitat topsoils were 75.9 µg/g for greenhouse and 87.0 µg/g for open field. For the cucumber fruits, the Zn concentration was 46.5 µg/g for greenhouse and 38.8 µg/g for open-field cultivation.

Khan et al. [54] concluded that potato cultivated on soil treated with sewage water was a potent threat to human health since Zn manifested toxicity after entering the food chain. Islam et al. [55] reported that the ranges of Cd and Ni were 3.90–13.0 and 104–443 µg/g, respectively, based on samples collected from urbanized industrial area in agricultural soils of Bangladesh. Eliku and Leta [56], based on samples from in Koka and Wonji farms (Ethiopia), reported that the overall results of the soil samples ranged from 44.4–88.5 µg/g for Zn. Later, Islam et al. [57] reported that the ranges of Cd and Ni were 0.60–13.0 and 3.9–36.0 µg/g, respectively, based on soil samples collected from vegetable-growing fields beside Paira River in the southern part of Bangladesh. Xue et al. [58] reported that the Zn level in sewage-irrigated soil (Baoding City, China) was the highest (154 µg/g), followed by Cu (35.1 µg/g), compared to those in the reference soil. This was attributed to long-term sewage irrigation that caused the elevated levels of Zn in the soil.

### 4.3. Correlations of Metals between Amaranthus and the Geochemical Fractions of the Habitat Topsoils

The current investigation showed no reasonable correlations for Zn pairwises. This could be expected, notwithstanding the root take-up of metal to the leaves, and environmental factors could impact the bioavailability and pollution of Zn in nearby vegetables [55]. Xu et al. [59] inferred that the soil metal bioavailability to the vegetables was subjected to the specific metal and vegetable species. Liu et al. [60] reported that the metal levels in vegetables and the associated soils were not strongly associated.

The positive and significant correlations for Cd, Fe, and Ni between the plant and their geochemical fractions in the habitat topsoils indicated that these metals in the environmental soil were considered readily and potentially bioavailable to the vegetables [61]. Fan et al. (2017) examined the correlations of PTM levels between the nursery vegetables and soil general properties (counting the geochemical parts). They concluded that the centralisations of Cd, Fe, and Ni in nursery vegetables were fundamentally correlated with convergences of various types of PTMs in nursery soil, demonstrating that higher levels Cd, Fe, and Ni levels in vegetables could be anticipated by the higher levels of metals in soils. Fan et al. [46] likewise reported that the levels of Ni in nursery leafy vegetables were profoundly (R = 0.85) related to the Ni bound to the geochemical part of natural matter and sulphides in the nursery soil. This shows that Ni in vegetables could be anticipated by the Ni concentrations in the natural matter and sulphides of the soil. Consequently, the present study showed that Ni bioaccumulation in the *Amaranthus* leaves and stems could be influenced by the Ni levels in the geochemical safe parts.

The distinction of soil bioavailability between the metals and the vegetables was essentially dependent on the levels of PTMs [15]. Roy and McDonald [62] reported that elevated Zn levels (1493 µg/g) were found in radish roots. They concluded that the joined dissolvable and interchangeable geochemical fractions of the surrounding soils could be utilised to assess the bioavailability of Zn for the plant species. However, Liu et al. [60] found that the metal levels in the vegetables were weakly related to those in the habitat topsoils.

### 4.4. Human Health Risk of Metals in Amaranthus

The THQ values for Cd, Fe, Ni, and Zn in the *A. viridis* were all below 1.00, indicating there were no non-carcinogenic risks of the six metals to the consumers, including children and adults.

Qureshi et al. [63] reported that the most noteworthy Fe level to purchasers’ consumption came from lettuce, which had multiple times higher content of this metal than any other vegetables. However, the current Fe THQ for *A. viridis* was below 1.0. Thus, the utilisation of *A. viridis* from Peninsular Malaysia would not cause any unfavourable (non-cancer-causing) impacts due to a high Fe exposure. Roy and Mcdonald [62] reported that carrot and lettuce could cause toxicological issues in children and adults, because of Zn accumulation. Zhang et al. [64] collected nursery surface soils (0–20 cm) and 30 vegetables from Kunming City (southwestern China), and analysed their Cd, Pb, Cu, Zn, As, Hg, and Cr contents. They found that there was no health risk of PTMs for children and adults.

Eliku and Leta [56] reported that the metal levels were 14.4 µg/g for Zn, in cabbage in Koka and Wonji farms (Ethiopia). They concluded that the consumption of these vegetables would not pose a human health risk. Based on the vegetables collected from Bogra District (Bangladesh), Islam et al. [65] demonstrated that the THQ value for Zn was under 1, showing that the consumers would not experience non-carcinogenic risks of Zn. However, Islam et al. [57] revealed that the THQ values of PTMs from all vegetables from Bangladesh were higher than 1, showing non-carcinogenic risks of PTMs to the consumers.

Lion and Olowoyo [66] reported that the THQ value was higher for Zn than Cu from all the plant parts of *Spinacia oleracea*, and these THQ values demonstrated that people were at health risk of Zn if they consumed spinach from these waste dump sites in and around Tshwane, South Africa. Wang et al. [67] revealed the THQ values of Zn in vegetables were under 1, while Hu et al. [52] concluded that the THQ values of Zn through vegetable utilisation were more than 1 for leafy vegetables from China, and the levels were higher for nursery vegetables than those from the open field.

### 4.5. Amaranthus as Phytoextractor of Ni and Zn

The use of other plants as phytoextractors of Ni and Zn as reported in the literature [68,69,70,71,72] supported the present finding of using *A. viridis*. For example, Favas et al. [68] studied the correlation between Ni concentration in the soil and its concentration in the plant (*Alyssum serpyllifolium*) in Portugal. In this study, the results showed that the phytoextraction process helped to concentrate Ni and Zn in the roots and stems. Phytoextraction, the absorption and accumulation of PTMs in the plant shoots and their removal from the treatment site through harvesting the plant parts, has been one of the many strategies for the phytoremediation of metal-polluted soils [24,73]. This method requires the uptake of pollutants from the plant roots and the translocation of the metals to the other parts (stems and leaves) of the plants [74]. This is followed by biomass harvest of the plant parts for safe disposal of the accumulated metals.

However, it should be noted that many abiotic factors could influence the efficiency of the phytoextraction processes, such as physico-chemical properties of the soil, metal bioavailability to the plants, metal speciation, climatic conditions, and the plant’s characteristics [75,76]. In theory, plants that act as phytoextractors could accumulate massive amounts of pollutants [75]. However, the suitability of a plant as a phytoextractor species for PTMs depends on the metal concentrations in the shoot (stems and leaves) and the shoot biomass. Being a non-hyperaccumulator, the phytoextraction approach that fits *Amaranthus* well is the relatively higher above ground biomass production due to its fast growth rate despite its lesser PTMs accumulation. This had also been reported in plants such as *Brassica* sp. [77].

### 4.6. Amaranthus as a Phytostabiliser of Cd, and Fe

The use of other plants as phytostabilisers of Cd and Fe, as reported in the literature [78,79,80], supported the present finding using *A. viridis*. For example, Drozdova et al. [77] studied the potential of and phytostabilisation of *Brassica campestris* for the concentrations of Cd, Cu, Ni, Pb, and Zn in the plant parts (leaves, roots, stems, and inflorescences). Their conclusion was based on the values of the BCF and TF. Mataruga et al. [81] reported that the BCF and TF values indicated that the elm (*Ulmus glabra*) was suitable for the phytostabilisation of Ni.

Yoon et al. [24] reported that the native plant (*Phyla nodiflora)* was suitable as a phytostabiliser of Zn, mainly attributed to *P. nodiflora*’s Zn accumulating abilities in its shoots (TF = 6.30). They also recommended *Gentiana pennelliana* as a good candidate for the phytostabilisation of Zn (BCF = 2.60) in polluted sites. Santos et al. [82] reported that the salt marsh *Tamarix africana* helped to stabilise the natural condition of soils, thus serving as a good phytostabilising agent for saline-contaminated soils. Varun et al. [83] reported that the weed *Abutilon indicum* displayed BCF > 1 at all concentrations. Therefore, it translocated most of the metals from its root.

According to Patra et al. [76], plants phytostabilised the soils by immobilising the pollutants in the rhizospheric region through adsorption or precipitation, thus preventing the pollutants from entering the environment as well as into the food chain of the ecosystem [74,75,84]. Metals accumulated by *A. viridis* would be channelled to the root tissues through phytostabilisation processes or transported through xylem vessels to the aerial parts of the plant. The various mechanisms in the roots of phytostabilisers involved adsorption, precipitation, and complexation [85].

### 4.7. Comparisons of Bioconcentration Factors of Potentially Toxic Metals in Amaranthus

Islam et al. [65], based on vegetables in the Bogra District (Bangladesh), reported the BCF value of Zn (0.39) from soil to the vegetables. Leafy vegetables had moderately higher BCF values of PTMs [47]. This might be due to more boundaries keeping substantial PTM transmission from soils to organic products than those to leaves [48]. The higher metal levels in the leafy vegetables could be ascribed to the generally enormous surface space of vegetable leaves. The low degrees of metals concentrated in the eatable portions of vegetables were ascribed to the low accumulation of them from the soil to the vegetables [55].

Jeddi and Chaieb [86] reported a high value of Zn BCF in *Erodium glaucophyllum*. Moreover, the higher TF values of Cd, Zn, and Fe in *E. glaucophyllum* shoots made it reasonable for their phytoextraction from the soil, while the lower TF values for Mn and Cu made this plant helpful for their phytostabilisation. Additionally, the good positive relationships of Mn, Pb, Cu, and Zn between habitat soils and *Erodium* organs might allow its potential use as a biomonitor of the PTMs. Pachura et al. [23] examined the impact of treating soil on the capacity to move Cd, Zn, Pb, and Ni from the soil to plant roots and afterwards to the above-ground portions by *Virginia fanpetals.* They found it had higher TF values for Cd and Zn than for Pb.

Since the BCF values for metals/EFLE in *Amaranthus* were seen to be higher than those for metals/SUM, metals were expected to show higher bioavailabilities to the plant by means of the EFLE fractions than in SUM of the soil. The higher BCF values (BCF_leaf/EFLE_, BCF_stem/EFLE_, and BCF_root/EFLE_) for Fe and Zn than those for other metals agreed with the findings of Liu et al. [61] and Qureshi et al. [63]. These outcomes likewise showed that Fe and Zn were effectively translocated to the leafy parts, while the movements of Cd and Ni from soils into the consumable parts of the vegetables encountered considerably more opposition. Subsequently, Fe was simpler than Ni to move from soil to the edible leaves of the plants. The BCF values of *A. viridis* found in the present study were supported by the findings of Liu et al. [61].

## 5. Conclusions

From the present study based on 11 sampling sites, it was found that the Cd concentrations in the leaves of *A. viridis* ranged from 0.45 to 2.18 µg/g dw (0.05–0.26 µg/g ww). The Fe concentrations in the leaves of *A. viridis* ranged from 74.8 to 535 µg/g dw (8.97–64.19 µg/g ww). The Ni concentrations (µg/g dw) in the leaves of *A. viridis* ranged from 2.02 to 7.45 µg/g dw (0.24–0.89 µg/g ww). The Zn concentrations in the leaves of *A. viridis* ranged from 65.2 to 521 µg/g dw (7.83–62.57 µg/g ww).

For Cd, significant (*p* < 0.05) and positive correlations were found in the pairwises for leaf–EFLE, stem–RES, stem–SUM, root–RES, and root–SUM. For Fe, significant (*p* < 0.05) and positive correlations were found in the pairwises for root–RES and root–SUM, while negative significant correlations (*p* < 0.05) were found for stem–OO and root–AR. For Ni, significant (*p* < 0.05) and positive correlations were found in the pairwises for leaf–RES and stem–RES. However, there were no significant (*p* > 0.05) correlations for all Zn pairwises. These positive relationships indicated the potential of *A. viridis* as a good biomonitor of Cd, Fe, and Ni pollution in the habitat topsoils.

In general, the THQ for Cd, Fe, Ni, and Zn in the *A. viridis* were below 1.00, indicating there were no non-carcinogenic risks of the four metals to the consumers, including children and adults. Nevertheless, routine monitoring and management of vegetable farms are recommended. Thus, with most of the values of BCF > 1.0 and TF > 1.0, *A. viridis* is a very promising phytoextraction agent of Ni and Zn. Additionally, with most of the values of BCF > 1.0 and TF < 1.0, *A. viridis* is a very promising phytostabiliser of Cd and Fe.

## Figures and Tables

**Table 1:** Information on the sampling sites of *Amaranthus viridis*, and their habitat topsoils from Peninsular Malaysia.

No	Sampling Sites	Sampling Date	GPS	Received Wastewater	Site Descriptions
1	Ara Kuda (Penang)	12 Sep 2017	N 05°27′09″	Yes	Agriculture area and palm oil plantation
			E 100°31′12″		
2	Kg Sitiawan (Perak)	14 Sep 2017	N 04°15′18″	Yes	Near coastal region and residential area
			E 100°42′27″		
3	Felda Taib Andak	4 Nov 2017	N 01°43′55″	No	Palm oil plantation
	(Johor)		E 103°38′23″		
4	Sikamat (N.Sembilan)	15 Oct 2017	N 02°46′24″	No	Residential area and road side
			E 101°59′28″		
5	Kuala Pegang	16 Sep 2017	N 05°39′13″	Yes	Agriculture area
	(Kedah)		E 100°54′35″		
6	Kg Raja (Terengganu)	17 Jan 2018	N 05°48′19″	No	Residential area and road side
			E 102°35′09″		
7	Kelaboran (Kelantan)	18 Jan 2018	N 06°11′14″	Yes	Residential area and road side
			E 102°10′25″		
8	Batu 12 Gombak (Selangor)	9 Oct 2017	N 03°15′46″	Yes	Residential area and road side
			E 101°43′22″		
9	Simpang Ampat	16 Oct 2017	N 02°26′44″	No	Residential area and highway
	(Melaka)		E 102°11′12″		
10	Kampung Tawar	21 Dec 2017	N 05°26′39″	Yes	Agriculture area
	(Perak)		E 101°07′13″		
11	Benta (Pahang)	14 Nov 2017	N 03°47′15″	Yes	Rubber plantation
			E 101°51′31″		

**Table 2:** Overall concentration (mean ± SE, µg/g dry weight) of Cd, Fe, Ni, and Zn in the geochemical fractions of the habitat topsoils of *Amaranthus viridis* collected from Peninsular Malaysia. Based on 11 sampling sites.

	EFLE	AR	OO	RES	SUM
Cd					
Min	0.18	0.24	0.26	1.12	2.11
Max	0.45	0.71	1.33	4.00	5.17
Mean	0.28	0.46	0.64	2.37	3.75
SE	0.03	0.05	0.09	0.27	0.30
Skewness	0.67	0.16	0.79	0.48	−0.04
Kurtosis	−0.49	−1.10	0.22	−0.71	−1.16
Fe					
Min	0.40	26.5	105	8084	8430
Max	1.39	216	244	58,216	58,375
Mean	0.73	143	175	25,513	25,832
SE	0.10	19.4	14.5	5178	5160
Skewness	0.96	−0.92	0.14	0.80	0.79
Kurtosis	−0.33	−0.29	−1.40	−0.78	−0.79
Ni					
Min	0.25	0.20	2.43	2.94	8.07
Max	1.70	4.27	9.53	14.4	24.8
Mean	0.99	1.16	5.24	7.07	14.5
SE	0.14	0.36	0.70	1.08	1.81
Skewness	0.26	1.74	0.36	1.02	0.67
Kurtosis	−0.93	2.32	−1.03	−0.12	−0.83
Zn					
Min	1.38	10.7	15.8	14.8	42.7
Max	5.90	57.6	93.5	117	246
Mean	2.75	34.2	51.7	56.7	145
SE	0.48	3.96	8.44	10.3	21.4
Skewness	1.33	0.25	0.13	0.71	0.40
Kurtosis	0.26	−0.18	−1.51	−0.91	−1.12

Note: EFLE = easily, freely leachable or exchangeable; AR = acid-reducible; OO = oxidisable-organic; RES = resistant; SUM = summation of the four geochemical fractions. Min = minimum; Max = maximum; SE = standard error.

**Table 3:** Correlation coefficients of concentrations log_10_(mean + 1) of Cd, Fe, Ni, and Zn between *Amaranthus viridis* (leaves, stems, and roots), and their habitat topsoils (geochemical fractions) (N = 11).

	EFLE	AR	OO	RES	SUM
Cd					
Leaf	**0.65**	−0.10	−0.18	0.32	0.24
Stem	0.48	−0.04	0.13	**0.58**	**0.57**
Root	0.55	0.46	0.34	**0.73**	**0.86**
Fe					
Leaf	0.20	−0.27	−0.37	0.32	0.32
Stem	0.17	−0.55	−**0.57**	0.48	0.48
Root	0.25	−**0.60**	−0.50	**0.69**	**0.68**
Ni					
Leaf	0.04	0.08	−0.20	**0.57**	0.24
Stem	0.08	0.10	0.01	**0.57**	0.34
Root	−0.53	−0.50	−0.02	−0.40	−0.35
Zn					
Leaf	0.31	0.21	−0.27	0.03	−0.05
Stem	0.33	0.28	−0.23	0.00	−0.04
Root	0.31	0.30	−0.08	0.14	0.09

Note: Values in bold are significantly correlated at *p* < 0.05. EFLE = easily, free, leachable, or exchangeable; AR- acid-reducible; OO = oxidisable–organic; RES = resistant; SUM = summation of all four geochemical fractions.

**Table 4:** Stepwise multiple linear regression analysis of potentially toxic metals concentrations (based on log_10_[mean + 1]) between the different parts of *Amaranthus viridis* (as dependent variables), and the geochemical fractions of the habitat topsoils (as independent variables) (N = 11).

Dependent Variables	Independent Variables Selected	R	R^2^
Cd-leaf	0.14 + 2.35 (EFLE) − 0.42 (OO)	0.74	0.55
Cd-stem	−0.12 + 0.45 (RES) + 1.21 (EFLE)	0.65	0.42
Cd-root	−0.16 + 0.81 (SUM)	0.86	0.73
Fe-leaf	3.73 − 0.72 (OO)	0.37	0.14
Fe-stem	2.53 − 0.79 (OO) + 0.23 (RES)	0.65	0.42
Fe-root	−0.69 + 0.72 (RES)	0.69	0.47
Ni-leaf	0.51 + 0.64 (RES) − 0.44 (OO)	0.71	0.51
Ni-stem	−0.03 + 0.49 (RES)	0.57	0.32
Ni-root	0.52 − 1.07 (EFLE) + 0.81 (OO) − 0.64 (AR)	0.78	0.60
Zn-leaf	No significant variable was selected.	-	-
Zn-stem	1.72 + 0.26 (EFLE) − 0.73 (OO) + 0.92 (AR)	0.63	0.40
Zn-root	No significant variable was selected.	-	-

Note: EFLE—easily, freely leachable or exchangeable; AR—acid-reducible; OO—oxidisable–organic; RES—resistant; SUM—summations of four geochemical fractions; those dependent variables were significantly (*p* < 0.05) influenced by the selected independent variables.

**Table 5:** Overall values of estimated daily intake (EDI, µg/kg wet weight/day), and the target hazard quotient (THQ, unitless) values of Cd, Fe, Ni, and Zn on the edible leaves of *Amaranthus viridis* (based on 11 sampling sites).

	EDI Child	THQ Child	EDI Adult	THQ Adult	EDI Child	THQ Child	EDI Adult	THQ Adult
	Zn	Zn	Zn	Zn	Fe	Fe	Fe	Fe
Min	8.53	0.028	3.85	0.013	9.78	0.014	4.41	0.006
Max	68.19	0.227	30.74	0.102	69.95	0.100	31.54	0.045
Mean	31.03	0.103	13.99	0.047	20.79	0.030	9.37	0.013
SD	7.10	0.024	3.20	0.011	5.10	0.007	2.30	0.003
Skewness	0.54	0.538	0.54	0.531	2.49	2.493	2.49	2.475
Kurtosis	−1.40	−1.398	−1.40	−1.411	4.92	4.925	4.92	4.869
	Cd	Cd	Cd	Cd	Ni	Ni	Ni	Ni
Min	0.06	0.059	0.03	0.027	0.26	0.013	0.12	0.006
Max	0.29	0.285	0.13	0.129	0.97	0.049	0.44	0.022
Mean	0.14	0.136	0.06	0.061	0.61	0.031	0.28	0.014
SD	0.02	0.018	0.01	0.008	0.07	0.004	0.030	0.002
Skewness	1.51	1.410	1.71	1.431	−0.08	−0.058	−0.08	−0.054
Kurtosis	2.16	1.948	2.57	1.995	−1.30	−1.294	−1.30	−1.292

Note: Min = minimum; Max = maximum; SE = standard error.

**Table 6:** Overall values of bioconcentration factors (BCF) of Cd, Fe, Ni, and Zn on the leaves, stems, and roots of *Amaranthus viridis* in Peninsular Malaysia (based on 11 sampling sites).

	BCF_leaf/EFLE_	BCF_leaf/SUM_	BCF_stem/EFLE_	BCF_stem/SUM_	BCF_root/EFLE_	BCF_root/SUM_
	Cd	Cd	Cd	Cd	Cd	Cd
Min	2.06	0.10	1.02	0.10	2.52	0.24
Max	5.70	0.51	5.73	0.37	7.06	0.51
Mean	3.81	0.29	2.91	0.21	5.36	0.38
SD	0.39	0.04	0.44	0.03	0.46	0.02
Skewness	0.18	0.35	0.39	0.41	−0.72	−0.25
Kurtosis	−1.34	−0.48	−0.58	−1.42	−0.66	0.59
	Fe	Fe	Fe	Fe	Fe	Fe
Min	97.09	0.00	37.2	0.00	153	0.00
Max	972.74	0.03	317	0.01	1277	0.03
Mean	253.70	0.01	103	0.00	485	0.01
SD	74.00	0.00	23.3	0.00	116	0.00
Skewness	2.56	1.79	2.08	1.64	1.18	0.93
Kurtosis	5.14	2.55	3.56	2.02	−0.02	0.00
	Ni	Ni	Ni	Ni	Ni	Ni
Min	2.13	0.14	0.51	0.02	0.73	0.05
Max	22.12	0.83	7.56	0.35	11.7	0.75
Mean	6.34	0.37	2.22	0.13	5.31	0.32
SD	1.74	0.07	0.61	0.03	1.09	0.06
Skewness	2.01	1.09	1.72	1.46	0.18	0.48
Kurtosis	3.20	−0.14	2.43	1.68	−1.07	−0.14
	Zn	Zn	Zn	Zn	Zn	Zn
Min	13.8	0.33	9.10	0.22	9.72	0.23
Max	206	5.09	80.8	2.00	101.07	2.18
Mean	93.5	2.02	52.7	1.12	59.10	1.21
SD	19.2	0.49	7.04	0.20	7.46	0.21
Skewness	0.65	0.64	−0.35	−0.18	−0.23	−0.15
Kurtosis	−0.90	−0.80	−0.79	−1.52	−0.16	−1.43

Note: Min = minimum; Max = maximum; SE = standard error.

**Table 7:** Overall values of translocation factors (TF) of Cd, Fe, Ni, and Zn on the leaves, stems, and roots of *Amaranthus viridis* in Peninsular Malaysia (based on 11 sampling sites).

	TF_leaf/root_	TF_stem/root_	TF_leaf/root_	TF_stem/root_
	Cd	Cd	Ni	Ni
Min	0.30	0.28	0.44	0.08
Max	1.23	0.86	5.34	2.23
Mean	0.76	0.53	1.77	0.62
SD	0.09	0.06	0.47	0.19
Skewness	0.15	0.22	1.22	1.71
Kurtosis	−0.74	−1.35	0.41	2.09
	Fe	Fe	Zn	Zn
Min	0.14	0.10	0.88	0.61
Max	0.94	0.38	2.61	1.04
Mean	0.59	0.24	1.47	0.89
SD	0.07	0.03	0.15	0.04
Skewness	−0.29	0.00	1.01	−0.80
Kurtosis	−0.49	−0.95	0.49	−0.63

Note: Min = minimum; Max = maximum; SE = standard error.

## Data Availability

Data is contained within the article or supplementary material.

## Supplementary Materials

The following are available online at https://www.mdpi.com/article/10.3390/biology11030389/s1, Table S1: Overall concentrations (mean ± SE, μg/g dry weight) of heavy metals (Cd, Fe, Ni, and Zn) in the leaves, stems and roots of Amaranthus viridis collected from Peninsular Malaysia; Table S2: Concentration (Mean ± SE, μg/g dry weight) of Cd in the geochemical fractions of the habitat topsoils of Amaranthus viridis collected from all sampling sites in Peninsular Malaysia; Table S3: Concentration (mean ± SE, μg/g dry weight) of Fe in the geochemical fractions of the habitat topsoils of Amaranthus viridis collected from all sampling sites in Peninsular Malaysia; Table S4: Concentration (mean ± SE, μg/g dry weight) of Ni in the geochemical fractions of the habitat topsoils of Amaranthus viridis collected from all sampling sites in Peninsular Malaysia; Table S5: Concentration (mean ± SE, μg/g dry weight) of Zn in the geochemical fractions of the habitat topsoils of Amaranthus viridis collected from all sampling sites in Peninsular Malaysia; Table S6: Values of estimated daily intake (EDI, μg/kg wet weight/day) and target hazard quotient (THQ, unitless) values of Cd, Fe, Ni and Zn on the edible leaves of Amaranthus viridis from all the sampling sites in Peninsular Malaysia; Table S7: Values of bioconcentration factors (BCF) of Cd, Fe, Ni and Zn on the leaves, stems and roots of Amaranthus viridis from all the sampling sites in Peninsular Malaysia; Table S8: Values of translocation factors (TF) of Cd, Fe, Ni and Zn on the leaves, stems and roots of Amaranthus viridis from all the sampling sites in Peninsular Malaysia.
