# Supplementary material for: Potentially Toxic Metals in the High-Biomass Non-Hyperaccumulating Plant Amaranthus viridis: Human Health Risks and Phytoremediation Potentials
Source: Biology (Basel). 2022 Mar 1;11(3):389. doi: 10.3390/biology11030389 (PMC8945204; doi:10.3390/biology11030389)
Supplement: Supplementary file 1 [file biology-11-00389-s001.zip › biology-1421844-supplementary.pdf]

Supplementary Materials

Potentially Toxic Metals in the High-Biomass Non-Hyperaccumulating Plant *Amaranthus viridis*: Human Health Risks and Phytoremediation Potentials

Chee Kong Yap [1,*], Aziran Yaacob [1], Wen Siang Tan [2,3], Khalid Awadh Al-Mutairi [4], Wan Hee Cheng [5], Koe Wei Wong [1], Franklin Berandah Edward [6], Mohamad Saupi Ismail [7], Chen Feng You [8], Weiyun Chew [9], Rosimah Nulit [1], Mohd. Hafiz Ibrahim [1], Bintal Amin [10] and Moslem Sharifinia [11]

S-1. Overall concentrations (mean ± SE, µg/g dry weight) of heavy metals (Cd, Fe, Ni, and Zn) in the leaves, stems and roots of *Amaranthus viridis* collected from Peninsular Malaysia. WW= converted to wet weight basis.

**Cd**	**Leaves**	**WW**	**Stems**	**WW**	**Roots**	**WW**
Min	0.45	0.05	0.28	0.02	0.70	0.08
Max	2.18	0.26	1.77	0.11	2.46	0.27
Mean	1.04	0.12	0.80	0.05	1.44	0.16
SE	0.14	-	0.15	-	0.15	-
Skewness	1.40	-	0.79	-	0.54	-
Kurtosis	1.93	-	-0.53	-	-0.27	-
Fe	Leaves	WW	Stems	WW	Roots	WW
Min	74.8	8.97	27.7	1.66	102	11.2
Max	535	64.2	174	10.5	988	109
Mean	159	19.1	64.9	3.90	320	35.2
SE	39.0	-	12.4	-	78.0	-
Skewness	2.49	-	1.93	-	1.65	-
Kurtosis	4.92	-	2.72	-	1.73	-
Ni	Leaves	WW	Stems	WW	Roots	WW
Min	2.02	0.24	0.37	0.02	1.12	0.12
Max	7.45	0.89	2.81	0.17	6.61	0.73
Mean	4.68	0.56	1.67	0.10	3.86	0.42
SE	0.56	-	0.26	-	0.56	-
Skewness	-0.06	-	-0.11	-	-0.17	-
Kurtosis	-1.29	-	-1.31	-	-1.09	-
Zn	Leaves	WW	Stems	WW	Roots	WW
Min	65.2	7.83	52.2	3.13	55.7	6.13
Max	521	62.6	341	20.5	357	39.3
Mean	237	28.5	134	8.02	148	16.3
SE	54.3	-	26.6	-	27.4	-
Skewness	0.54	-	1.16	-	1.09	-
Kurtosis	-1.40	-	0.68	-	0.48	-

Note: The dry weights of leaves, roots and stems were converted into wet weight (WW) basis by using conversion factors of 0.12, 0.11, and 0.06, respectively.
Min= Minimum; Max= Maximum; SE= Standard error.

S-2. Concentration (Mean ± SE, µg/g dry weight) of Cd in the geochemical fractions of the habitat topsoils of *Amaranthus viridis* collected from all sampling sites in Peninsular Malaysia.

No.	EFLE			AR			OO			RES			SUM	NR (%)	RES (%)
1	0.18	±	0.00	0.27	±	0.02	0.32	±	0.00	2.22	±	0.01	2.99	2.64	74.16
2	0.26	±	0.01	0.45	±	0.02	0.81	±	0.06	2.52	±	0.01	4.03	3.98	62.40
3	0.29	±	0.01	0.35	±	0.06	0.26	±	0.01	4.00	±	0.01	4.90	4.55	81.69
4	0.37	±	0.02	0.48	±	0.07	0.68	±	0.04	3.64	±	0.01	5.17	5.14	70.43
5	0.45	±	0.02	0.55	±	0.01	0.81	±	0.05	2.97	±	0.00	4.78	4.71	62.15
6	0.26	±	0.01	0.55	±	0.02	1.33	±	0.03	1.65	±	0.00	3.80	3.42	43.56
7	0.22	±	0.01	0.67	±	0.01	0.83	±	0.05	2.59	±	0.00	4.31	4.19	60.03
8	0.18	±	0.01	0.71	±	0.01	0.42	±	0.02	2.00	±	0.00	3.32	3.27	60.38
9	0.21	±	0.01	0.24	±	0.02	0.54	±	0.06	1.12	±	0.00	2.11	2.28	52.85
10	0.36	±	0.01	0.45	±	0.01	0.67	±	0.05	1.44	±	0.00	2.92	2.94	49.34
11	0.28	±	0.02	0.29	±	0.01	0.34	±	0.01	1.97	±	0.00	2.88	2.80	68.45

Note: EFLE= easily, freely, leachable or exchangeable; AR= acid-reducible; OO- oxidisable-organic; RES= resistant; SUM= summation of the four geochemical fractions; NR= nonresistant.

S-3. Concentration (mean ± SE, µg/g dry weight) of Fe in the geochemical fractions of the habitat topsoils of *Amaranthus viridis* collected from all sampling sites in Peninsular Malaysia.

No.	EFLE			AR			OO			RES			SUM	NR (%)	RES (%)
1	0.78	±	0.11	205	±	3.90	232	±	4.91	9733	±	489	10171	4.31	95.69
2	0.40	±	0.06	163	±	3.55	182	±	3.12	8084	±	130	8430	4.10	95.90
3	0.77	±	0.07	27.9	±	1.51	131	±	10.7	58216	±	2097	58375	0.27	99.73
4	1.31	±	0.40	26.5	±	1.36	105	±	3.33	49731	±	2567	49864	0.27	99.73
5	0.67	±	0.00	144	±	6.64	229	±	20.9	32528	±	359	32902	1.14	98.86
6	1.39	±	0.54	153	±	2.09	138	±	2.41	15826	±	638	16118	1.81	98.19
7	0.55	±	0.06	119	±	7.12	133	±	2.88	20035	±	108	20288	1.25	98.75
8	0.82	±	0.12	172	±	12.3	142	±	7.77	18215	±	986	18530	1.70	98.30
9	0.43	±	0.11	150	±	10.0	179	±	2.44	16697	±	217	17026	1.93	98.07
10	0.43	±	0.06	199	±	3.44	205	±	2.14	11016	±	429	11421	3.54	96.46
11	0.45	±	0.08	216	±	2.77	244	±	16.5	40566	±	1134	41027	1.12	98.88

Note: EFLE= easily, freely, leachable or exchangeable; AR= acid-reducible; OO- oxidisable-organic; RES= resistant; SUM= summation of the four geochemical fractions; NR= nonresistant.

S-4. Concentration (mean ± SE, µg/g dry weight) of Ni in the geochemical fractions of the habitat topsoils of *Amaranthus viridis* collected from all sampling sites in Peninsular Malaysia.

No.	EFLE			AR			OO			RES			SUM	NR (%)	RES (%)
1	0.65	±	0.03	0.5	±	0.01	2.43	±	0.19	7.03	±	0.26	10.6	33.73	66.27
2	0.72	±	0.02	0.2	±	0.02	5.08	±	0.05	8.95	±	0.04	14.94	40.13	59.87
3	0.25	±	0.05	0.43	±	0.02	2.61	±	0.58	5.69	±	0.75	8.99	36.68	63.32
4	0.85	±	0.01	0.3	±	0.01	3.21	±	0.12	3.71	±	0.19	8.07	54.05	45.95
5	0.76	±	0.02	0.92	±	0.01	7.51	±	0.04	4.84	±	0.27	14.03	65.52	34.48
6	0.73	±	0.01	1.75	±	0.02	6.71	±	0.21	5.63	±	0.14	14.82	62.03	37.97
7	1.53	±	0.24	0.45	±	0.05	3.67	±	0.05	5.62	±	0.03	11.27	50.1	49.9
8	0.95	±	0.04	0.63	±	0.03	3.63	±	0.1	2.94	±	0.25	8.15	63.91	36.09
9	1.57	±	0.01	4.27	±	0.12	6.26	±	0.09	12.71	±	1.02	24.81	48.78	51.22
10	1.7	±	0.02	1.58	±	0.07	9.53	±	0.01	6.27	±	0.09	19.08	67.16	32.84
11	1.16	±	0.06	1.78	±	0.12	7.01	±	0.13	14.39	±	0.32	24.34	40.89	59.11

Note: EFLE= easily, freely, leachable or exchangeable; AR= acid-reducible; OO- oxidisable-organic; RES= resistant; SUM= summation of the four geochemical fractions; NR= nonresistant.

S-5. Concentration (mean ± SE, µg/g dry weight) of Zn in the geochemical fractions of the habitat topsoils of *Amaranthus viridis* collected from all sampling sites in Peninsular Malaysia.

No.	EFLE			AR			OO			RES			SUM	NR (%)	RES (%)
1	2.32	±	0.32	24.6	±	0.49	28.1	±	1.84	43.4	±	0.47	98.4	55.88	44.12
2	2.36	±	0.31	34.4	±	0.64	20.4	±	1.07	38.2	±	1.51	95.4	59.96	40.04
3	1.45	±	0.21	24.9	±	0.31	76.1	±	2.57	56.6	±	4.16	159	64.43	35.57
4	2.39	±	0.23	32.9	±	0.83	93.5	±	1.36	117	±	2.65	246	52.44	47.56
5	2.83	±	0.22	30.9	±	0.52	25.9	±	0.75	24.1	±	0.73	83.8	71.25	28.75
6	5.73	±	0.88	53.4	±	1.68	80.9	±	1.60	102	±	3.88	242	57.79	42.21
7	1.47	±	0.20	40.3	±	1.42	57.8	±	0.86	39.1	±	0.46	139	71.81	28.19
8	5.90	±	0.21	57.6	±	2.53	81.4	±	0.81	100	±	2.12	246	58.97	41.03
9	2.04	±	0.14	36.4	±	1.45	52.5	±	3.34	47.2	±	1.51	138	65.86	34.14
10	2.35	±	0.42	29.7	±	0.91	36.4	±	1.92	40.7	±	0.46	109	62.70	37.30
11	1.38	±	0.26	10.7	±	0.22	15.8	±	1.42	14.8	±	0.50	42.7	65.41	34.59

Note: EFLE= easily, freely, leachable or exchangeable; AR= acid-reducible; OO- oxidisable-organic; RES= resistant; SUM= summation of the four geochemical fractions; NR= nonresistant.

S-6. Values of estimated daily intake (EDI, µg/kg wet weight/day) and target hazard quotient (THQ, unitless) values of Cd, Fe, Ni and Zn on the edible leaves of *Amaranthus viridis* from all the sampling sites in Peninsular Malaysia.

Cd	Children		Adults	
	EDI	THQ	EDI	THQ
1	0.13	0.131	0.06	0.059
2	0.11	0.114	0.05	0.052
3	0.13	0.134	0.06	0.061
4	0.18	0.182	0.08	0.082
5	0.29	0.285	0.13	0.129
6	0.08	0.079	0.04	0.036
7	0.06	0.059	0.03	0.027
8	0.14	0.136	0.06	0.061
9	0.14	0.141	0.06	0.064
10	0.12	0.119	0.05	0.054
11	0.11	0.112	0.05	0.050
Fe	Children		Adults	
	EDI	THQ	EDI	THQ
1	14.74	0.021	6.65	0.009
2	12.09	0.017	5.45	0.008
3	18.17	0.026	8.19	0.012
4	21.92	0.031	9.88	0.014
5	21.06	0.030	9.50	0.014
6	21.55	0.031	9.72	0.014
7	69.95	0.100	31.54	0.045
8	10.39	0.015	4.68	0.007
9	12.15	0.017	5.48	0.008
10	9.78	0.014	4.41	0.006
11	16.89	0.024	7.61	0.011
Ni	Children		Adults	
	EDI	THQ	EDI	THQ
1	0.83	0.042	0.38	0.019
2	0.53	0.027	0.24	0.012
3	0.72	0.036	0.33	0.016
4	0.87	0.044	0.39	0.020
5	0.40	0.020	0.18	0.009
6	0.27	0.014	0.12	0.006
7	0.48	0.024	0.22	0.011
8	0.26	0.013	0.12	0.006
9	0.80	0.040	0.36	0.018
10	0.58	0.029	0.26	0.013
11	0.97	0.049	0.44	0.022
Zn	Children		Adults	
	EDI	THQ	EDI	THQ
1	46.30	0.154	20.87	0.070
2	63.48	0.212	28.62	0.095
3	12.79	0.043	5.77	0.019

4	11.93	0.040	5.38	0.018
5	20.74	0.069	9.35	0.031
6	10.36	0.035	4.67	0.016
7	8.53	0.028	3.85	0.013
8	68.19	0.227	30.74	0.102
9	29.67	0.099	13.38	0.045
10	58.20	0.194	26.24	0.087
11	11.13	0.037	5.02	0.017

Note: The dry weight basis of heavy metal concentrations in the edible leaves of *Amaranthus viridis* was converted to wet weight basis by using a conversion factor of 0.12.

S-7. Values of bioconcentration factors (BCF) of Cd, Fe, Ni and Zn on the leaves, stems and roots of *Amaranthus viridis* from all the sampling sites in Peninsular Malaysia.

Cd	$BCF_{leaf/EFLE}$	$BCF_{leaf/SUM}$	$BCF_{stem/EFLE}$	$BCF_{stem/SUM}$	$BCF_{root/EFLE}$	$BCF_{root/SUM}$
1	5.48	0.34	4.38	0.27	6.63	0.41
2	3.40	0.22	5.73	0.36	6.67	0.42
3	3.59	0.21	3.51	0.21	5.78	0.34
4	3.78	0.27	2.92	0.21	5.49	0.39
5	4.83	0.46	3.92	0.37	5.44	0.51
6	2.35	0.16	1.90	0.13	5.17	0.35
7	2.06	0.10	2.37	0.12	6.84	0.35
8	5.70	0.31	2.00	0.11	7.06	0.39
9	5.04	0.51	3.27	0.33	4.34	0.44
10	2.56	0.31	1.02	0.12	3.04	0.37
11	3.09	0.30	1.02	0.10	2.52	0.24
Fe	$BCF_{leaf/EFLE}$	$BCF_{leaf/SUM}$	$BCF_{stem/EFLE}$	$BCF_{stem/SUM}$	$BCF_{root/EFLE}$	$BCF_{root/SUM}$
1	144.90	0.011	56.03	0.004	153.34	0.012
2	229.09	0.011	114.43	0.005	304.37	0.015
3	179.72	0.002	132.06	0.002	1277.18	0.017
4	128.11	0.003	55.03	0.001	195.68	0.005
5	240.91	0.005	85.24	0.002	411.10	0.008
6	118.67	0.010	37.20	0.003	265.06	0.023
7	972.74	0.026	316.72	0.009	1121.71	0.030
8	97.09	0.004	55.35	0.002	193.97	0.009
9	218.24	0.005	112.45	0.003	594.20	0.015
10	173.67	0.007	64.39	0.002	236.12	0.009
11	287.51	0.003	103.99	0.001	577.20	0.006
Ni	$BCF_{leaf/EFLE}$	$BCF_{leaf/SUM}$	$BCF_{stem/EFLE}$	$BCF_{stem/SUM}$	$BCF_{root/EFLE}$	$BCF_{root/SUM}$
1	9.80	0.60	2.35	0.14	7.26	0.44
2	5.68	0.27	3.07	0.15	9.18	0.44
3	22.12	0.62	7.56	0.21	11.72	0.33
4	7.85	0.83	3.31	0.35	7.08	0.75
5	4.05	0.22	1.43	0.08	7.03	0.38
6	2.86	0.14	0.51	0.02	6.53	0.32
7	2.41	0.33	0.56	0.08	0.73	0.10
8	2.13	0.25	0.56	0.07	3.81	0.44

9	3.88	0.25	1.62	0.10	0.73	0.05
10	2.59	0.23	1.04	0.09	2.31	0.21
11	6.42	0.31	2.41	0.12	2.00	0.10
Zn	$BCF_{leaf/EFLE}$	$BCF_{leaf/SUM}$	$BCF_{stem/EFLE}$	$BCF_{stem/SUM}$	$BCF_{root/EFLE}$	$BCF_{root/SUM}$
1	152.74	3.60	79.53	1.87	79.39	1.87
2	205.58	5.09	80.83	2.00	78.73	1.95
3	67.45	0.61	39.43	0.36	57.26	0.52
4	38.09	0.37	25.56	0.25	41.91	0.41
5	56.11	1.89	40.70	1.37	44.04	1.49
6	13.82	0.33	9.10	0.22	9.72	0.23
7	44.36	0.47	52.33	0.56	50.29	0.53
8	88.40	2.12	57.83	1.39	60.56	1.45
9	111.01	1.64	65.98	0.98	79.04	1.17
10	189.34	4.08	80.29	1.73	101.07	2.18
11	61.48	1.99	48.47	1.57	48.12	1.56

S-8. Values of translocation factors (TF) of Cd, Fe, Ni and Zn on the leaves, stems and roots of *Amaranthus viridis* from all the sampling sites in Peninsular Malaysia.

	$TF_{leaf/root}$	$TF_{stem/root}$	$TF_{leaf/root}$	$TF_{stem/root}$
No.	Cd		Fe	
1	0.83	0.66	0.94	0.37
2	0.51	0.86	0.75	0.38
3	0.62	0.61	0.14	0.10
4	0.69	0.53	0.65	0.28
5	0.89	0.72	0.59	0.21
6	0.45	0.37	0.45	0.14
7	0.30	0.35	0.87	0.28
8	0.81	0.28	0.50	0.29
9	1.16	0.75	0.37	0.19
10	0.84	0.33	0.74	0.27
11	1.23	0.41	0.50	0.18
No.	Ni		Zn	
1	1.35	0.32	1.92	1.00
2	0.62	0.33	2.61	1.03
3	1.89	0.65	1.18	0.69
4	1.11	0.47	0.91	0.61
5	0.58	0.20	1.27	0.92
6	0.44	0.08	1.42	0.94
7	3.29	0.77	0.88	1.04
8	0.56	0.15	1.46	0.95
9	5.34	2.23	1.40	0.83
10	1.12	0.45	1.87	0.79
11	3.21	1.21	1.28	1.01
